# Gastric Emphysema Related to Percutaneous Endoscopic Gastrostomy After Two-Stage Esophagectomy: A Report of Two Cases

**DOI:** 10.7759/cureus.67626

**Published:** 2024-08-23

**Authors:** Toshiyuki Moriuchi, Yasuhiro Shirakawa, Yuki Katsura, Michihiro Ishida, Yasuhiro Choda

**Affiliations:** 1 Department of Surgery, Hiroshima City Hiroshima Citizens Hospital, Hiroshima, JPN

**Keywords:** diabetes mellitus, delayed gastric emptying, post-esophagectomy, portal venous gas, gastric emphysema

## Abstract

Patients with esophageal cancer who have severe complications such as diabetes sometimes require two-stage surgery. Herein, we describe two cases of gastric emphysema that were treated at our facility after the patients had previously undergone minimally invasive esophagectomy as the first-stage surgical treatment of esophageal cancer. Case 1: A 72-year-old man with a history of diabetes mellitus (DM) was diagnosed with esophageal cancer and an esophageal obstruction and subsequently underwent percutaneous endoscopic gastrostomy (PEG) placement followed by neoadjuvant chemoradiotherapy. The treatment efficacy was good; once the tumor was deemed resectable, the patient underwent robot-assisted minimally invasive esophagectomy and cervical esophagostomy placement as the first stage of surgical treatment. The patient had a good postoperative course and was discharged on postoperative day (POD) 10. However, on POD 16, he returned to the hospital with abdominal distension. Computed tomography (CT) revealed gastric emphysema and hepatic portal vein gas. Conservative treatment was initiated as there were no signs of peritoneal irritation. An upper gastrointestinal (GI) series revealed delayed gastric emptying (DGE); therefore, replacement of the PEG with a percutaneous endoscopic gastrojejunostomy (PEG-J) was necessary. On POD 42, the patient underwent reconstructive surgery as the second-stage surgical treatment of esophageal cancer. Case 2: A 74-year-old man had a history of DM, chronic renal failure, and PEG placement for dysphagia caused by left recurrent nerve palsy after thoracic aortic aneurysm surgery. The patient underwent a thoracoscopic esophagectomy with cervical esophagostomy placement as the first-stage surgical treatment of esophageal cancer. On POD 6, the patient developed abdominal distension, his CT showed gastric emphysema. An upper GI series was performed, which showed DGE. After conservative treatment and improvement in his general condition, the patient underwent a jejunostomy placement on POD 30. Both patients developed gastric emphysema related to PEG placement after undergoing esophagectomy as the first-stage surgical treatment of esophageal cancer. Additionally, both patients had a history of DM. Gastric emphysema, which is thought to be caused by increased intragastric pressure due to postoperative DGE, developed within 30 days of undergoing minimally invasive esophagectomy in both patients. Therefore, the rate of nutrient administration and symptoms should be carefully monitored during the postoperative management of patients with these characteristics.

## Introduction

Gastric emphysema is a rare disease characterized by the presence of gas within the gastric wall, which can be effectively diagnosed using computed tomography (CT). It is important to differentiate gastric emphysema from emphysematous gastritis [1,2]. Both are distinct diseases, as gastric emphysema is caused by a non-infectious mechanism, while emphysematous gastritis is caused by gas-producing bacteria [3]. Additionally, the prognosis of each condition is markedly different, gastric emphysema has a better prognosis, while emphysematous gastritis has a much higher mortality rate [4]. Most cases of gastric emphysema improve with conservative management, including bowel rest through fasting, the intravenous administration of proton pump inhibitors, and the use of antibiotics, depending on the case [5]. Some patients with diabetes mellitus (DM) have been reported to develop delayed gastric emptying (DGE) due to dysfunction of the autonomic nervous system, which regulates gastrointestinal (GI) motility [6]. Surgical treatment for esophageal cancer is considered highly invasive compared to that for other GI cancers. Depending on the patient’s condition, a two-stage surgery may be selected to balance the invasiveness.

Herein, we describe two cases of postoperative gastric emphysema in patients with esophageal cancer. Both patients underwent two-stage esophagectomy and were diagnosed with gastric emphysema via CT after experiencing abdominal distention following an increase in tube feedings through their percutaneous endoscopic gastrostomy (PEG) tubes. Both patients were also diagnosed with DGE through upper GI fluoroscopy. The cases presented herein emphasize the necessity of managing postoperative care and monitoring symptoms in patients with esophageal cancer with PEG placement, particularly when adjusting tube feeding rates.

## Case presentation

Case 1

**Figure 1:**
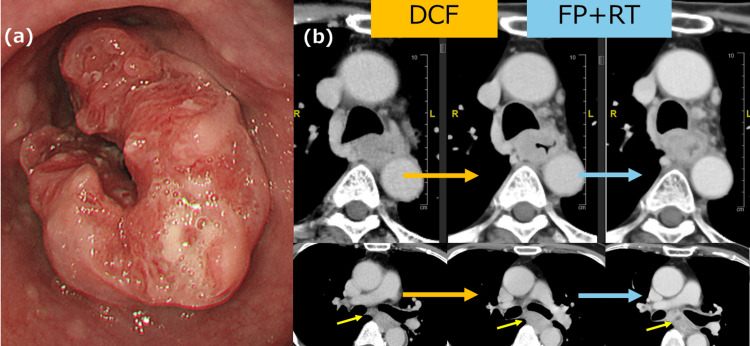
EGD and CT findings (a): Type 2 esophageal SCC, occupying two-thirds of the circumference of the upper middle esophagus. (b): One course of chemotherapy, DCF, slightly increased the size of the tumor; however, chemoradiotherapy (FP+RT) decreased the size of the tumor. The lymph nodes in the tracheal bifurcation demonstrated enlargement. Post-chemoradiotherapy imaging revealed a low-density region within the lymph nodes, consistent with necrosis (yellow arrow). EGD: esophagogastroduodenoscopy; CT: computed tomography; SCC: squamous cell carcinoma; DCF: docetaxel, 5-fluorouracil, and cisplatin; FR: 5-fluorouracil and cisplatin

A 72-year-old man with a history of DM and hypertension was diagnosed with esophageal squamous cell carcinoma (SCC) based on the results of an esophagogastroduodenoscopy (EGD), which revealed a type 2 lesion occupying two-thirds of the circumference of his upper middle esophagus (Figure 1a). The HbA1c National Glycohemoglobin Standardization Program (NGSP) was 7.1%. CT of the chest/abdomen/pelvis showed metastasis to one lymph node in the mediastinum. Therefore, the patient was diagnosed with Ut/Mt (23-28 cm), involving 2/3 of the circumference and located on the right wall, 5 cm in size, type 2 SCC, cT3r, cN1, cM0, and cStage IIIb according to the Japanese Classification of Esophageal Cancer, 12th Edition. The patient underwent PEG placement due to the presence of a tumor-induced stricture and impaired fluid passage. A single cycle of docetaxel, 5-fluorouracil, and cisplatin (DCF) was administered as neoadjuvant chemotherapy; however, the tumor increased in size; therefore, we opted to switch to two courses of chemoradiotherapy, 5-fluorouracil and cisplatin (FP) and 46 Gy. The tumor shrunk in response to the change in treatment and was subsequently deemed resectable. The boundary between the tumor and the surrounding tissue subsequently became clear. The effect of the treatment was stable disease (SD) (Figure 1b).

**Figure 2:**
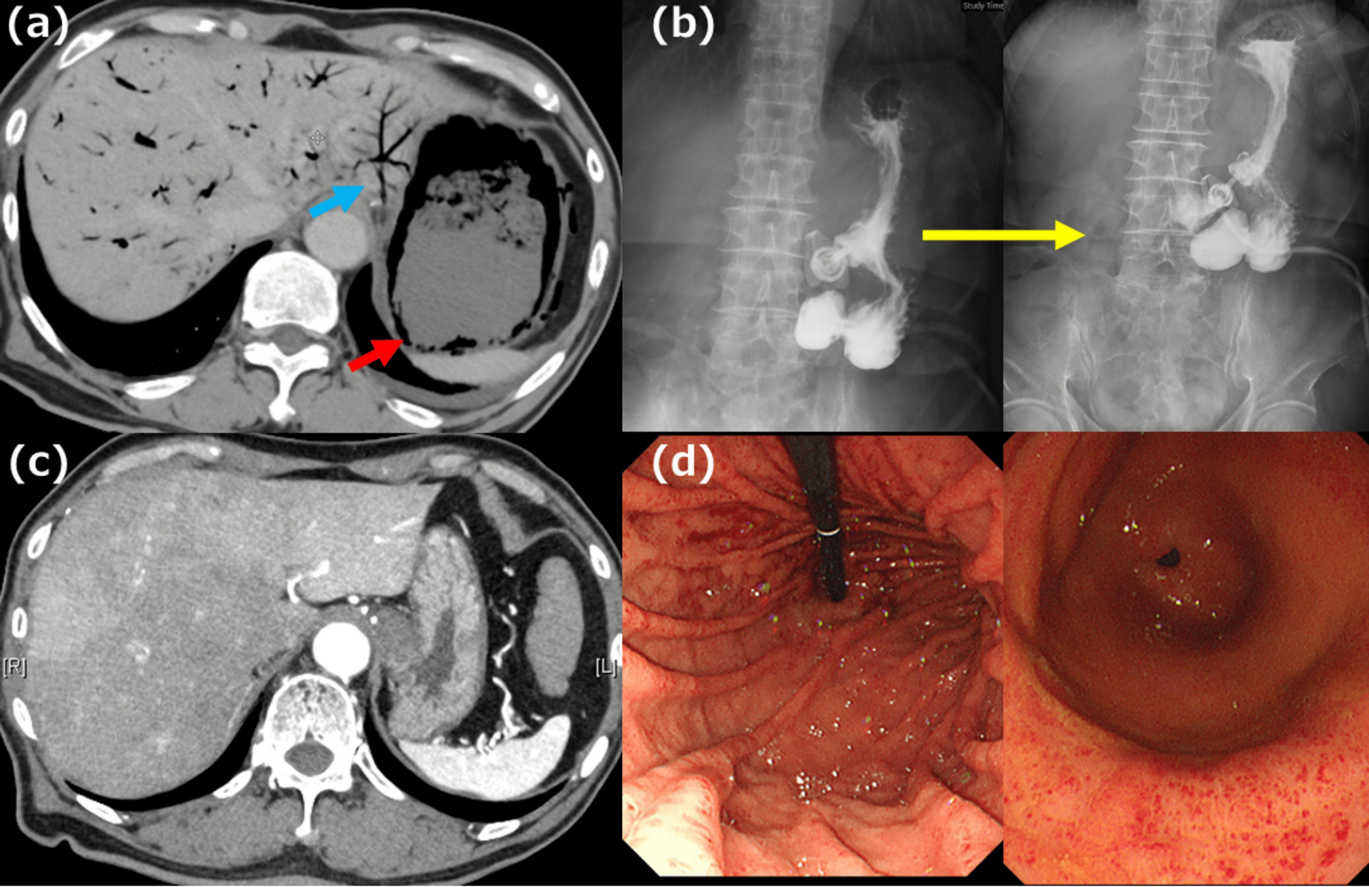
CT, upper GI series, and EGD findings in Case 1 (a) CT showed gastric emphysema (red arrow) and hepatic portal vein gas (blue arrow). (b) Four hours later, Gastrografin🄬 iodinated contrast did not pass the pylorus, indicating DGE. (c) After the sixth day of conservative treatment, gastric emphysema, and hepatic portal vein gas disappeared. (d) There is no erosion of the gastric mucosa and punctate redness is observed in the gastric antrum. No ischemic findings were observed. GI: gastrointestinal; CT: computed tomography; EGD: esophagogastroduodenoscopy; DGE: delayed gastric emptying

**Figure 3:**
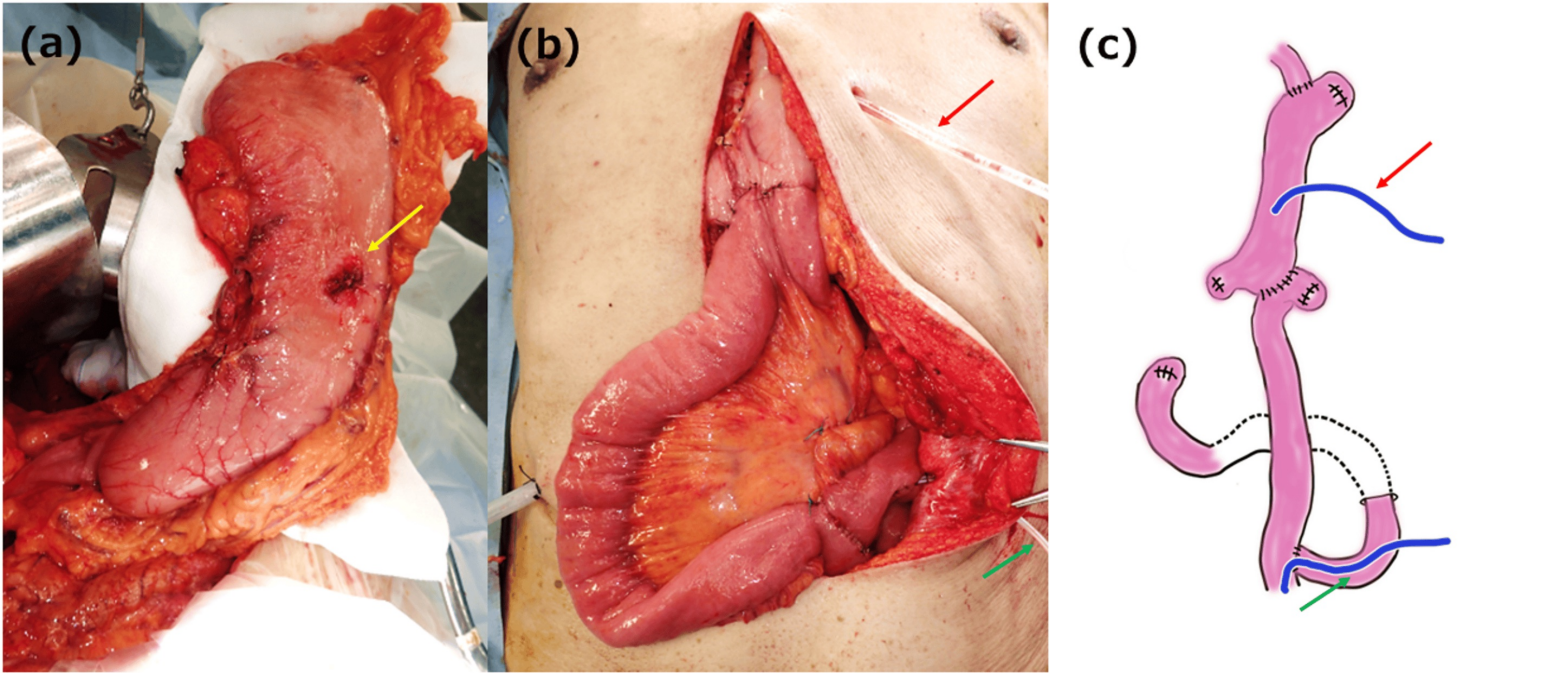
Operative findings in Case 1 (a) No sclerotic changes are observed in the stomach. The yellow arrow points to the PEG hole. (b,c) Gastric conduit reconstruction was performed under the skin route with Roux-en-Y anastomosis. The red arrow points to the decompression gastric tube. The green arrow points to the jejunostomy tube. Figure 3c was created by the author. PEG: percutaneous endoscopic gastrostomy

To maximize the effectiveness of the treatment while minimizing the extent of surgical intervention and its associated risks and complications, we opted for a two-stage surgical approach, the first of which was a robot-assisted minimally invasive esophagectomy with cervical esophagostomy placement. The patient’s postoperative pathological diagnoses were: Ut/Mt, 3 cm; type 5b; moderately differentiated SCC; CRT-pT2, INFb, Ly0, V0, pIM0, pPM0, pDM0, pRM0; multiple primary carcinomas (none); CRT-Grade 2; pN0 (0/16); sM0; CRT-fStage II. The patient’s postoperative course progressed well. The patient was transferred to another hospital on postoperative day (POD) 10 for recovery until the reconstruction surgery. At our hospital, he had been receiving a polymeric formula (Lakfia🄬) at a rate of 80 mL/hr, totaling 1600 mL/1600 kcal per day. At the receiving hospital, the feeding rate was increased to >100 mL/hr, with no specific focus on the rate of administration. However, on POD 16, the patient returned to our hospital with complaints of abdominal distention, and his CT revealed gastric emphysema and hepatic portal vein gas (Figure 2a). As there was no sign of peritoneal irritation and the inflammatory response was minimal, we opted for conservative treatment (fasting, opening the PEG tube for decompression, proton pump inhibitors, and antibiotics). On day 6 of re-hospitalization, an upper GI series under fluoroscopy showed DGE (Figure 2b). In contrast, CT showed that both the gastric emphysema and hepatic portal vein gas had resolved (Figure 2c). Therefore, the PEG had to be replaced with a PEG-J for the new nutrition route. Moreover, EGD showed no evidence of erosion of the mucosal surface (Figure 2d). On POD 42 after the first surgery, the patient underwent reconstructive surgery, which was the second stage of the two-stage surgery. No gastric wall sclerosis was observed (Figure 3a); therefore, we decided to preserve the stomach. The surgical technique involved the under-the-skin route with gastric conduit reconstruction, Roux-en Y anastomosis, and jejunostomy (Figure 3b, 3c). The patient was discharged on POD 24, following the second-stage surgery. The patient received a course of nivolumab as postoperative adjuvant chemotherapy and is currently recurrence-free at three months.

Case 2

**Figure 4:**
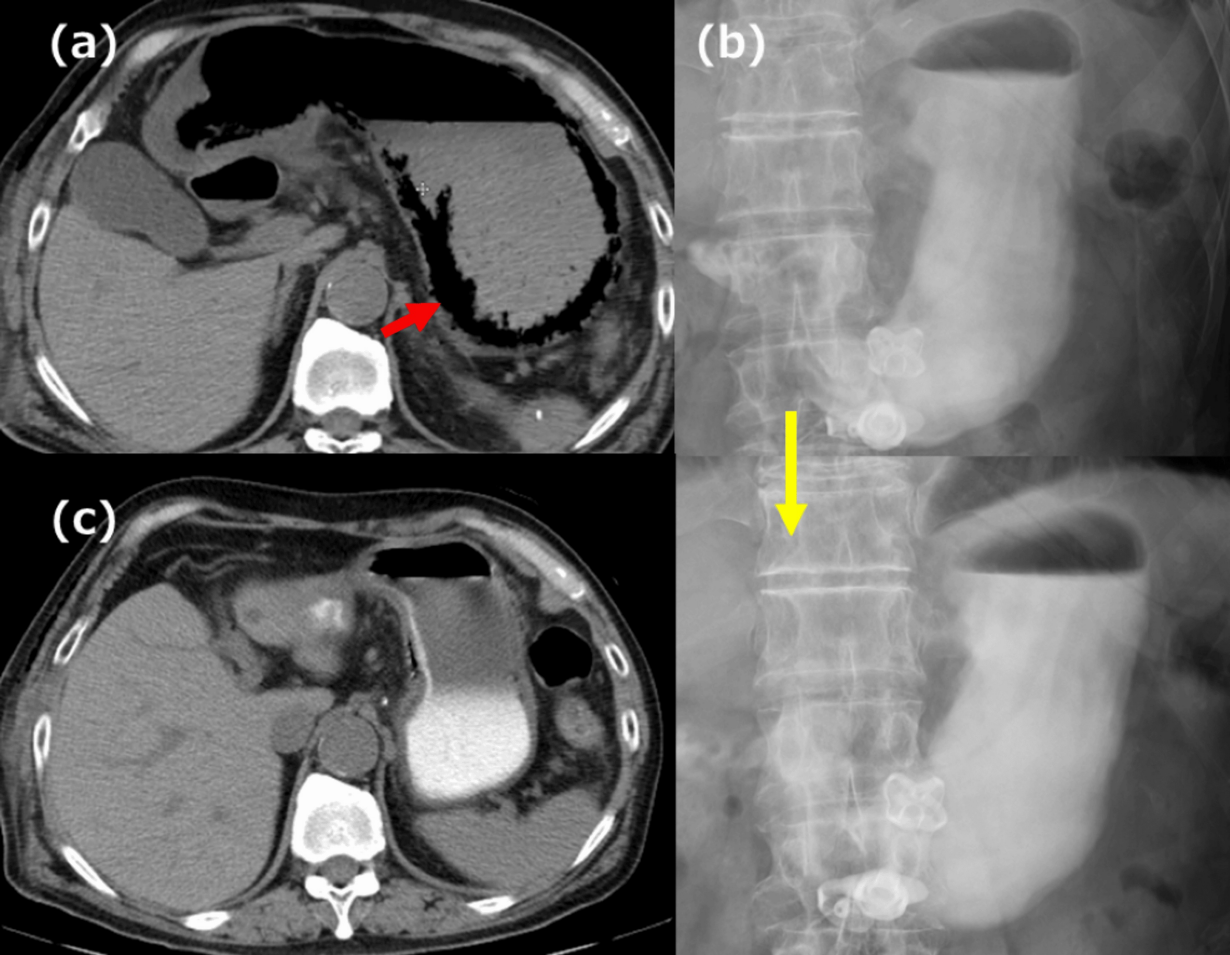
CT and upper GI series findings in Case 2 (a) CT showed gastric emphysema (red arrow). (b) Four hours later, Gastrografin🄬 iodinated contrast did not pass the pylorus, indicating DGE. (c) After the sixth day of conservative treatment, the gastric emphysema had resolved. CT: computed tomography; GI: gastrointestinal; DGE: delayed gastric emptying

A 74-year-old man with a history of DM, chronic renal failure, and PEG placement for dysphagia caused by left recurrent nerve palsy following post-surgical repair of a thoracic aortic aneurysm presented to our hospital. His HbA1c (NGSP) was 6.2%. The patient was diagnosed with esophageal SCC based on the results of an EGD, and a CT of the chest/abdomen/pelvis scan showed no lymph node or distal metastases. His preoperative clinical diagnosis was Mt/Lt (31-35 cm), involving 1/3 of the circumference and located on the right wall, 4 cm in size, type 0-Ⅱc SCC, cT1b-SM2, cN0, cM0, and cStage I. We opted for a two-stage esophagectomy. The patient underwent a thoracoscopic esophagectomy with cervical esophagostomy placement. On POD 2, he was started on a polymeric formula (Lakfia🄬) at a rate of 40 mL/hr. The patient complained of abdominal discomfort after the initial administration; therefore, the feeding rate was slowly increased. When administering a polymeric formula (Lakfia🄬) at a rate of 60 mL/hr on POD 6, he complained of abdominal distension, and a CT of the chest/abdomen/pelvis revealed gastric emphysema (Figure 4a). In light of the absence of peritoneal irritation, we opted for conservative treatment (fasting, opening the PEG tube for decompression, proton pump inhibitors). On POD 12, an upper GI series showed DGE and the patient’s CT revealed resolution of the gastric emphysema (Figure 4b, 4c). After the patient’s general condition improved, he underwent a jejunostomy on POD 30. Initially, reconstructive surgery was planned as the second-stage surgery; however, the patient never recovered swallowing function due to recurrent nerve palsy, even with rehabilitation. Hence, reconstructive surgery was not performed. The patient is currently recurrence-free; however, his DGE has not improved since the surgery.

## Discussion

When gas is detected within the layers of the gastric wall, the differential diagnoses are gastric emphysema and emphysematous gastritis [1,2]. Gastric emphysema has a noninfectious etiology and is classified into three types: traumatic, obstructive, and pulmonary. Traumatic gastric emphysema results from damage to the gastric mucosa by external factors; obstructive gastric emphysema arises from the migration of gas within the gastric wall due to increased intragastric pressure caused by GI obstruction; and pulmonary gastric emphysema is caused by gas from the lungs migrating into the gastric wall [1]. In the cases described herein, gastric emphysema was thought to have stemmed from the migration of gas into the gastric wall through the PEG site as a result of the elevated intragastric pressure caused by the DGE. Gastric emphysema generally has a good prognosis with appropriate treatments. Conversely, emphysematous gastritis results from an infection in which gas-producing bacteria result in the formation of emphysema within the gastric wall. This condition is severe and potentially fatal [3,4]. The microorganisms implicated in emphysematous gastritis include Streptococcus, *Escherichia coli*, Enterobacter, Clostridium, *Pseudomonas aeruginosa*, *Staphylococcus aureus*, Candida, and Mucor [7].

Due to its abundant blood supply and extensive collateral circulation, the stomach is not particularly susceptible to ischemia; therefore, necrosis of the stomach rarely occurs. In fact, prior studies have reported that ligation of all major gastric arteries does not result in stomach necrosis unless the veins are also ligated [8]. However, although rare, there have been reports of gastric emphysema progressing to gastric wall necrosis due to elevated intragastric pressure, which impairs venous return [9]. Therefore, when a patient has gastric emphysema, it is important for them to fast and to start decompression immediately following diagnosis. There have been reports of deaths due to gastric emphysema related to PEG; therefore, the patient’s condition should be closely monitored throughout the course following PEG placement.

**Figure 5:**
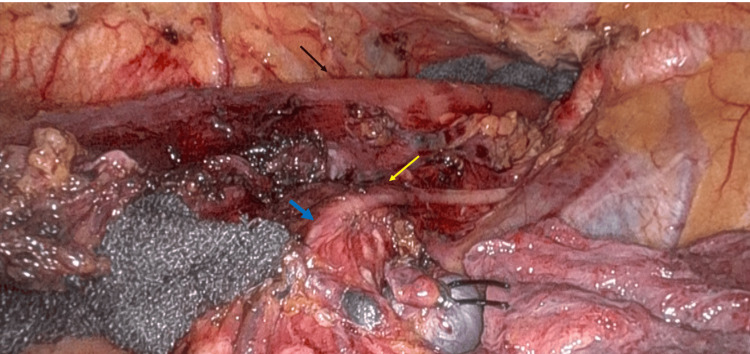
Operative findings in case 2 The black arrow points to the esophagus. The yellow arrow points to the right vagus nerve. The vagus nerve is transected at the level of the tracheal bifurcation during radical esophagectomy. The blue arrow indicates the cut-off edge of the right vagus nerve.

Diabetes-induced GI motility and sensory disturbances are thought to result from disorders of the enteric nervous system, interstitial cells of Cajal, and autonomic nervous system, which controls GI motility. These disturbances are reportedly caused by the interaction of a variety of factors, such as oxidative stress, autoimmunity, endothelial dysfunction, microflora, and microribonucleic acid (miRNA) [10]. Hyperglycemia in the stomach is thought to impair gastric contractions and result in DGE [6]. Iwamuro et al. reported that DM, nasogastric tubes, and PEG placement were the most common underlying causes of gastric emphysema and hepatic portal vein gas [5]. Therefore, patients with PEG placement and DM are more likely to develop gastric emphysema due to DGE. Additionally, the vagus nerve is always severed during radical surgery for esophageal cancer (Figure 5). Given that both patients presented herein showed marked postoperative DGE, we believe that vagotomy may further reduce gastric peristalsis in patients with DM who have undergone surgical resection for esophageal cancer treatment.

Excessively rushed administration of tube feedings is dangerous and requires appropriate perioperative management. In fact, concerning case 2, the patient reported abdominal symptoms post-administration and the onset of gastric emphysema might have been averted if an upper GI fluoroscopy had been conducted earlier. Thus, it is important to promptly evaluate the presence of DGE using upper GI fluoroscopy in patients whose feeding rate and symptoms are suspicious for DGE to prevent the development of gastric emphysema.

## Conclusions

Herein, we reported two cases of gastric emphysema related to PEG placement resulting from DGE following radical esophagectomy. To the best of our knowledge, this is the first report of such a case. In high-risk patients with DM and PEG placement who undergo two-stage esophagectomy, a postoperative impairment of gastric peristalsis may occur. Gastric emphysema can prolong hospitalization and result in further harm to patients. Therefore, early detection of symptoms and appropriate management of feeding rates are essential throughout the two-stage surgical treatment for esophageal cancer patients with severe complications.

## References

[1] Agha FP (1984). Gastric emphysema: an etiologic classification. Australas Radiol.

[2] Kussin SZ, Henry C, Navarro C, Stenson W, Clain DJ (1982). Gas within the wall of the stomach report of a case and review of the literature. Dig Dis Sci.

[3] López-Medina G, Castillo Díaz de León R, Heredia-Salazar AC, Hernández-Salcedo DR (2014). Gastric emphysema a spectrum of pneumatosis intestinalis: a case report and literature review. Case Rep Gastrointest Med.

[4] Watson A, Bul V, Staudacher J, Carroll R, Yazici C (2017). The predictors of mortality and secular changes in management strategies in emphysematous gastritis. Clin Res Hepatol Gastroenterol.

[5] Iwamuro M, Takenaka R, Toyokawa T (2024). Endoscopic and clinical features of gastric emphysema. Sci Rep.

[6] Camilleri M (2007). Diabetic gastroparesis. N Engl J Med.

[7] Sharma P, Akl EG (2016). A combination of intramural stomach and portal venous air: conservative treatment. J Community Hosp Intern Med Perspect.

[8] Babkin BP, Armour JC, Webster DR (1943). Restoration of the functional capacity of the stomach when deprived of its main arterial blood supply. Can Med Assoc J.

[9] Nakao A, Isozaki H, Iwagaki H, Kanagawa T, Takakura N, Tanaka N (2000). Gastric perforation caused by a bulimic attack in an anorexia nervosa patient: report of a case. Surg Today.

[10] Yarandi SS, Srinivasan S (2014). Diabetic gastrointestinal motility disorders and the role of enteric nervous system: current status and future directions. Neurogastroenterol Motil.

